# Double Ventricular Response with Aberrant Conduction Leading to Ventricular Dysfunction

**DOI:** 10.1007/s00246-024-03506-9

**Published:** 2024-05-06

**Authors:** Paul W. Warren, Adam W. Powell, Timothy Knilans, Chad Connor, Shankar Baskar

**Affiliations:** 1 The Heart Institute, Cincinnati Children’s Hospital Medical Center, 3333 Burnett Ave., MLC 2003, Cincinnati, OH 45229-3026 USA; 2 Department of Pediatrics, University of Cincinnati College of Medicine, Cincinnati, OH USA

**Keywords:** Left ventricular dysfunction, Dual AV nodal physiology, Dual atrioventricular nodal physiology, Atrioventricular nodal reentry tachycardia, Dual atrioventricular nodal non-re-entrant tachycardia, Double ventricular response

## Abstract

Double ventricular response (DVR), where a single P wave results in two QRS complexes, is a rare presentation of dual AV node physiology. It has been associated with ventricular dysfunction in the setting of incessant tachycardia. We present the case of an otherwise healthy adolescent who had frequent DVR without tachycardia leading to left ventricular dysfunction. Slow pathway modification led to a significant reduction in ectopy and normalization of ventricular function. This highlights that DVR without tachycardia might lead to ventricular dysfunction in pediatric patients. Slow pathway modification with reduction of ectopy may be sufficient to restore ventricular function.

## Introduction

Irregular heart rhythms in children are a common reason for cardiology referral, and there is rarely a pathologic process discovered [1]. Dual atrioventricular (AV) node physiology is a physiologic finding present in up to 10% of the normal population. The usual pathologic presentation of dual AV node physiology is AV nodal reentrant tachycardia (AVNRT). Double ventricular response (DVR), where a single P wave leads to two QRS complexes due to conduction via the fast and slow pathway, is a much rarer presentation of dual AV nodal physiology. Continuous DVR may lead to dual AV nodal non-reentrant tachycardia (DAVNNRT), which is being increasingly recognized [2, 3]. Similar to incessant AVNRT leading to left ventricular systolic dysfunction; DAVNNRT has been associated with ventricular dysfunction in the adult population [4, 5]. We present the rare case of an otherwise healthy adolescent who had frequent DVR without DAVNNRT or AVNRT leading to left ventricular dysfunction.

## Case Report

**Fig. 1:**
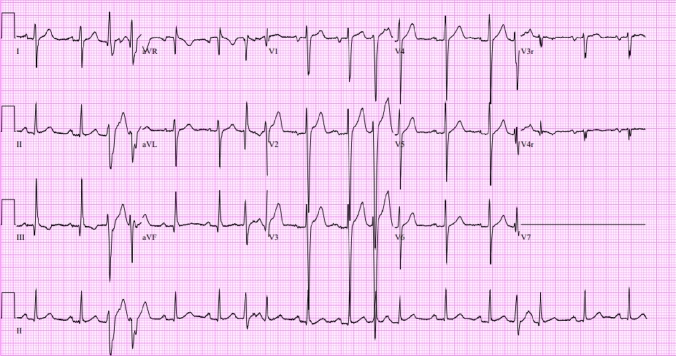
The initial ECG of a 13-year-old asymptomatic patient with an irregular rhythm. Wide QRS complexes were thought to be secondary to DVR with aberration, premature junctional beats with aberration, or premature ventricular complexes. Narrow QRS complexes were thought to be secondary to DVR conducted normally versus premature junctional beats

A previously healthy, athletic 13-year-old male (121 kg) was referred to cardiology clinic after the pediatrician auscultated an irregular rhythm on exam. He had no cardiac symptoms. His physical exam was remarkable for an irregular rhythm with occasional extra systoles auscultated. Initial ECG demonstrated multiple relatively narrow premature QRS complexes of different morphologies (Fig. 1). An echocardiogram was performed which revealed normal cardiac structure but mildly depressed left ventricular systolic function (Ejection fraction [EF]: 48%).

**Fig. 2:**
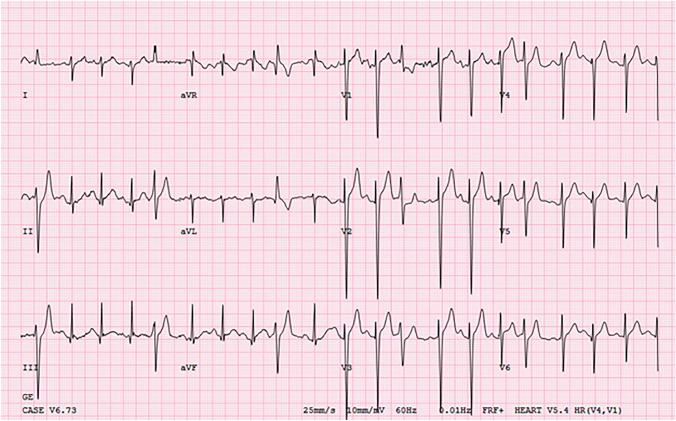
ECG during exercise test noting premature beats with narrow QRS complexes that were identical to the conducted beats without preceding atrial activity and did not disturb the atrial mechanism. There were also frequent premature beats with wide QRS complexes of varying morphology

A Holter monitor showed frequent extra systoles with multiple QRS morphologies without preceding P waves and episodes of non-sustained supraventricular tachycardia (SVT) without a clear P wave during the tachycardia (total ectopy burden: 23%, mostly aberrated beats). For further evaluation of initially suspected complex ventricular ectopy, an exercise test was performed. This was remarkable for premature beats with premature narrow QRS complexes prior to exercise that were identical to the conducted beats without preceding atrial activity and did not disturb the atrial mechanism (Fig. 2). There were also frequent premature beats with wide QRS complexes of varying morphology. During later stages of exercise there was sinus tachycardia without premature beats.

**Fig. 3:**
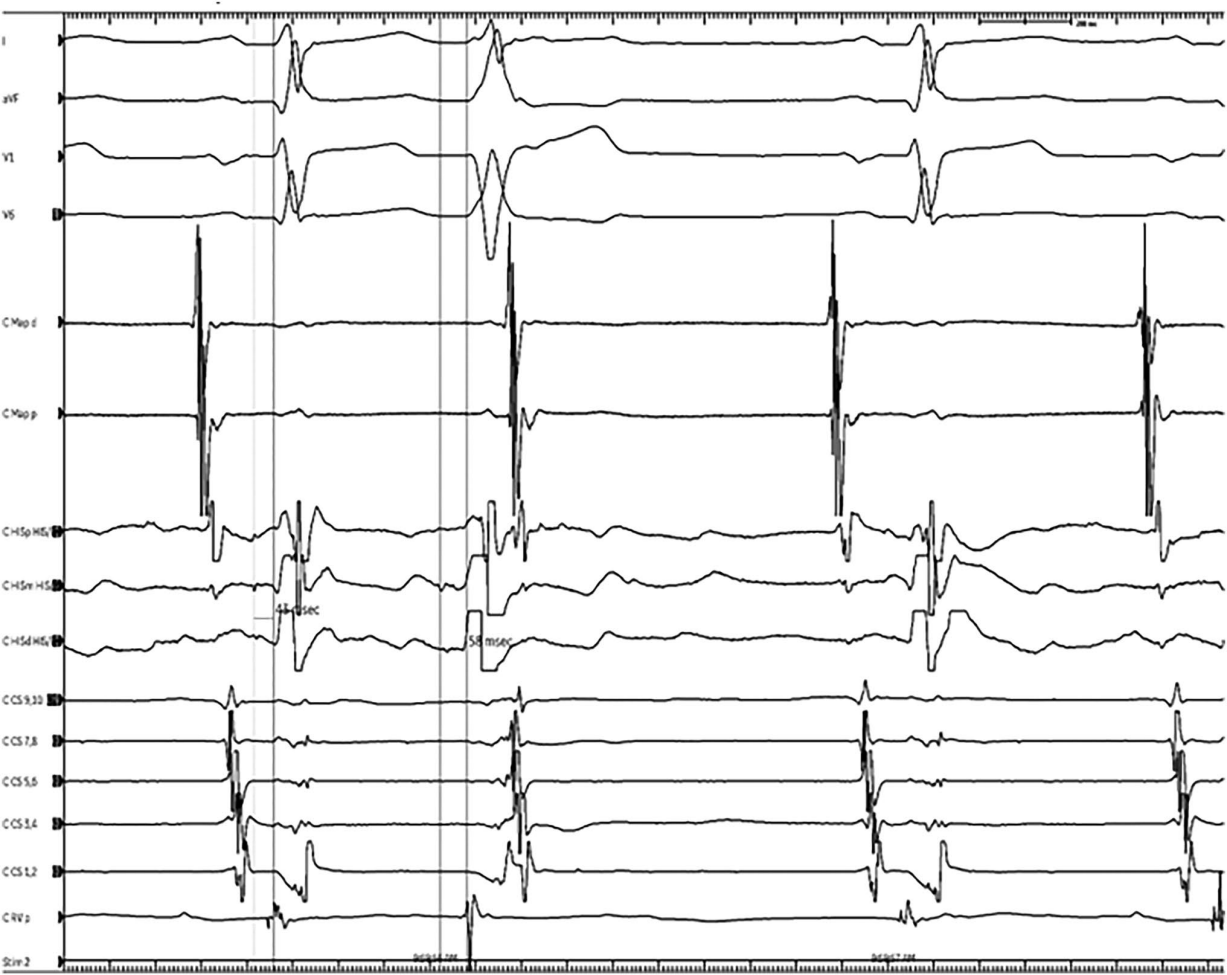
EP Study: Frequent 1:2 conduction was demonstrated. Here, an episode of 1:2 response with aberrant conduction is noted with prolonged HV interval on the aberrant beat

The differential diagnosis for the narrow QRS premature beats was thought to be intermittent DVR (1:2 conduction with dual AV nodal physiology) versus less likely premature junctional beats. The wide QRS premature beats were hypothesized to either represent DVR or junctional beats conducted with aberration versus multi-morphic premature ventricular contractions. A cardiac MRI did not show any evidence of myocardial scar or inflammation (no delayed late gadolinium enhancement with normal T1 and T2 values of the ventricular myocardium) and mildly depressed LV (EF: 44%) and RV function (EF: 43%) without regional wall motion abnormalities. Given the suspicion that DVR with frequent aberrant conduction was the underlying etiology of ventricular dysfunction, a comprehensive electrophysiology study (EPS) was performed (Fig. 3).

During the EPS, the presence of DVR was confirmed by noting His-bundle electrogram with the same activation sequence and unipolar polarity preceding both the narrow and wide beats. Furthermore, there was a prolonged HV interval in the presence of wide complex premature beats confirming that these were indeed due to aberrant conduction. There were also episodes of typical AV nodal echo without inducible SVT.

Slow pathway potentials were noted close to the level of the roof of coronary sinus ostium. We hence opted to perform slow pathway modification. At the end of the series of cryo lesions, there was complete suppression of DVR. Following this we tested for 1 h on and off isoproterenol infusion. This demonstrated persistence of slow pathway conduction but demonstrated evidence of modification by having no AV nodal echo while off isoproterenol and only rare episodes of DVR. Given the marked decrease in the frequency of DVR, the procedure was concluded.

In follow-up after his ablation, he was asymptomatic. On a Holter 2 weeks after the procedure, he was noted to have continued DVR with aberrant conduction though they occurred less frequently (total ectopy burden: 13%). At subsequent follow up after 8 months his Holter demonstrated an increase in total ectopy burden to 24% but his left ventricular function was now normal (EF: 57%). He was partaking in football training at the time of the Holter and it was hypothesized that the Holter might not have represented predominant resting state. At repeat follow up 3 years from the time of ablation his Holter demonstrated a total ectopy burden of 3% (mostly DVR conducted with aberration), significantly less than his initial presentation, and normal left ventricular function (EF: 60%). He remains asymptomatic and is active in sports.

## Discussion

We presented the case of a healthy adolescent with an irregular rhythm who was found to have DVR with resulting biventricular systolic dysfunction. To our knowledge, this is the first reported case of a pediatric patient that developed ventricular dysfunction in the setting of DVR who did not also have a significant burden of tachycardia. While slow pathway modification did not lead to complete elimination of dual AV node physiology, it did result in a significant reduction in the proportion of aberrated beats, which suggests that the presence of dual AV node physiology was responsible for the ectopy burden and dysfunction. This is further supported by the normalization of ventricular function following slow pathway modification and reduction in aberrancy on follow-up Holter monitoring.

Dual AV node physiology is not a rare entity [6, 7]. The presence of it provides a possible mechanism for, but is not necessarily predictive of, the development of AVNRT [3, 8, 9]. A less common phenomenon associated with dual AV node physiology is DAVNNRT where a single atrial beat results in two ventricular beats, i.e. 1:2 conduction, at a tachycardic rate [10]. There are two reported cases in pediatric patients, a 5-year-old and 16-year-old, who had DAVNNRT with incessant tachycardia; however, they had a notable absence of ventricular dysfunction [11, 12]. Our patient’s presentation is interesting in that while he has DVR, he has no significant tachycardia burden and yet developed diminished cardiac function. This case illustrates that DVR in the absence of tachycardia may predispose even pediatric patients to ventricular dysfunction, which can be improved following ablation. This is an isolated report, and studies with a reasonable number of patients and longer follow up are necessary to confirm these preliminary hypotheses.

## Conclusions

DVR might lead to complex ectopy and aberrancy which can lead to ventricular dysfunction in pediatric patients. Slow pathway modification with reduction in DVR may be sufficient in restoration of ventricular function, rather than the absolute need for slow pathway elimination.

## Abbreviations

AV: Atrioventricular
AVNRT: Atrioventricular nodal reentry tachycardia
DAVNNRT: Dual atrioventricular nodal non-re-entrant tachycardia
DVR: Double ventricular response
EF: Ejection fraction
EPS: Electrophysiology study
SVT: Supraventricular tachycardia
